# Adherence to Secondary Prevention Measures after Acute Myocardial Infarction and Its Impact on Patient Outcome—A Nationwide Perspective

**DOI:** 10.3390/jcm13164964

**Published:** 2024-08-22

**Authors:** Andreas Hammer, Hana Sinkovec, Marko Todorovic, Florian Katsch, Walter Gall, Georg Duftschmid, Georg Heinze, Alexander Niessner, Patrick Sulzgruber

**Affiliations:** 1 Division of Cardiology, Department of Internal Medicine II, Medical University of Vienna, 1090 Vienna, Austria; 2 Section for Clinical Biometrics, Center for Medical Statistics, Informatics and Intelligent Systems, Medical University of Vienna, Spitalgasse 23, 1090 Vienna, Austria; 3 Center for Medical Statistics, Informatics and Intelligent Systems, Department of Medical Information Management, Medical University of Vienna, 1090 Vienna, Austria; 4 2nd Department of Medicine with Cardiology and Intensive Care Medicine, Vienna Healthcare Group Clinic Landstrasse, 1030 Vienna, Austria

**Keywords:** acute coronary syndrome, cardiac rehabilitation, high-intensity statins, dual antiplatelet therapy, adherence, acute myocardial infraction

## Abstract

**Objectives:** Secondary prevention is crucial for reducing morbidity and mortality in patients following acute myocardial infraction (MI). However, adherence to cardiac rehabilitation (CR) and pharmacotherapy remains suboptimal despite strong guideline recommendations. This study investigated the adherence to CR, dual antiplatelet therapy (DAPT), and statins following acute MI and evaluated their impact on patient outcomes from a nationwide perspective in Austria. **Methods:** In this national observational study, all patients diagnosed with acute MI, defined as STEMI or NSTEMI, between April 2011 and August 2015 in Austria were included. Patient characteristics and comorbidities were derived from the Austrian national health insurance system using ICD-10 codes. Adherence to CR, high-intensity statins, and DAPT was assessed based on health insurance records and pharmacy prescription submissions. Cox Regression hazard analysis was used to explore the impact of non-adherence to CR on mortality. **Results:** Among 16,518 acute MI patients, only 13.4% adhered to the recommended CR programs, which was associated with a significantly lower risk of mortality (adjusted hazard ratio [HR] 0.73; 95% CI: 0.54–0.98; *p* = 0.036). In contrast, 66.4% of 23,240 patients did not comply with high-intensity statin therapy, correlating with an increased mortality risk (adjusted HR 1.16; 95% CI: 1.06–1.25; *p* < 0.001). Furthermore, among 22,331 patients analyzed for DAPT adherence, only 29.3% followed the guidelines, yet this adherence was linked to a 21% reduction in mortality over the observation period (adjusted HR 0.79; 95% CI: 0.72–0.88; *p* < 0.001). **Conclusions:** This nationwide study reveals alarmingly low adherence to CR and secondary preventive medications among acute MI patients, which is significantly linked to higher mortality rates. Enhanced efforts to promote awareness and adherence are crucial, involving structured referrals and personalized follow-ups to improve patient outcomes. Addressing these gaps through comprehensive healthcare strategies could substantially enhance cardiovascular health.

## 1. Introduction

Secondary prevention after acute myocardial infraction (MI) mirrors a key element in the reduction in morbidity and mortality in this highly vulnerable patient population [1]. In particular, cardiac rehabilitation (CR) represents a comprehensive and multifactorial therapy approach in secondary prevention and has consistently demonstrated itself as one of the most advantageous therapeutic interventions for reducing future cardiovascular events and effectively modifying cardiovascular risk factors [2]. CR is typically divided into three phases. Phase I occurs during the hospital stay, focusing on early mobilization and initial education [3]. Phase II, lasting three to six weeks, is conducted on an outpatient basis under medical supervision, emphasizing physical training and education. Phase III is a long-term maintenance phase where patients continue their rehabilitation independently, supported by periodic follow-up. The program includes medical assessments, physical training, patient education, psychosocial support, nutritional counseling, and adherence to pharmacotherapy. To foster adherence to pharmacotherapy, the program incorporates educational sessions where patients learn about the importance of their medications and how to manage them effectively and are encouraged to adhere to their prescribed treatment plans. Adherence to this intervention has been shown to decrease mortality, hospital readmissions, and healthcare costs and to improve exercise capacity, quality of life, and psychological well-being [2,4,5,6]. Despite recommendations of current clinical practice guidelines (class of recommendation: I; level of evidence: A), referral to a designated CR program after acute MI seems often suboptimal, with strong regional varieties across countries and municipal/rural regions [7,8].

Another important aspect in secondary prevention after acute MI is adherence to prescribed pharmacotherapy (i.e., statins, beta-blockers, renin–angiotensin system acting agents, and antiplatelet therapy). In particular, lipid-lowering therapy—via high-intensity statins (i.e., atorvastatin 40–80 mg q.d. or rosuvastatin 20–40 mg q.d.)—proved to be one of the most beneficial therapeutic approaches for the reduction in re-events [7,9]. Additionally, antiplatelet therapy is a critical cardioprotective factor across various phases of acute MI, encompassing preclinical loading to post-interventional or long-term therapy. It is widely recognized that acute MI patients are at an elevated risk of experiencing subsequent ischemic events, which further amplifies the mortality rate with each additional occurrence. In this regard, dual antiplatelet therapy (DAPT) serves as a fundamental component in the management of both ST elevation myocardial infarction (STEMI) and non-ST elevation myocardial infarction (NSTEMI) [7,9].

However, there is a lack of comprehensive epidemiological data on adherence to recommended CR following acute MI, despite its potential importance in preventing fatal cardiac adverse events. Moreover, the adherence to high-potency statins and DAPT is crucial in mitigating adverse outcomes in these patients. Importantly, adherence to these pharmacotherapies is frequently correlated with participation in CR. Consequently, our objective was to examine the adherence to CR, DAPT, and the utilization of statins after acute MI and assess their influence on patient outcome from a nationwide perspective in Austria.

## 2. Methods

Within this population-based national observation, all patients presenting with acute MI between 04/2011 and 8/2015 in Austria were enrolled. Acute MI was defined in accordance with the guidelines of the European Society of Cardiology as STEMI or NSTEMI. Patient characteristics and co-morbidities were assessed via the Austrian national health insurance system and elucidated according to ICD10 definitions. Adherence to recommended CR was investigated according to health insurance documentation and was defined as joining a designated rehabilitation program within 1 year after the index event. Adherence to high-intensity statins was investigated according to handing in prescriptions for rosuvastatin (20–40 mg) and atorvastatin (40–80 mg) at local pharmacies. Similarly, the adherence to DAPT was evaluated by assessing the submission of prescriptions for aspirin and P2Y_12_ inhibitors at local pharmacies. Patients were followed prospectively until the primary study endpoint (=mortality) was reached. Cox Regression hazard analysis was used to investigate the impact of non-adherence to CR on patient outcome and was adjusted for a comprehensive subset of confounders within the multivariate model.

## 3. Results

**Figure 1:**
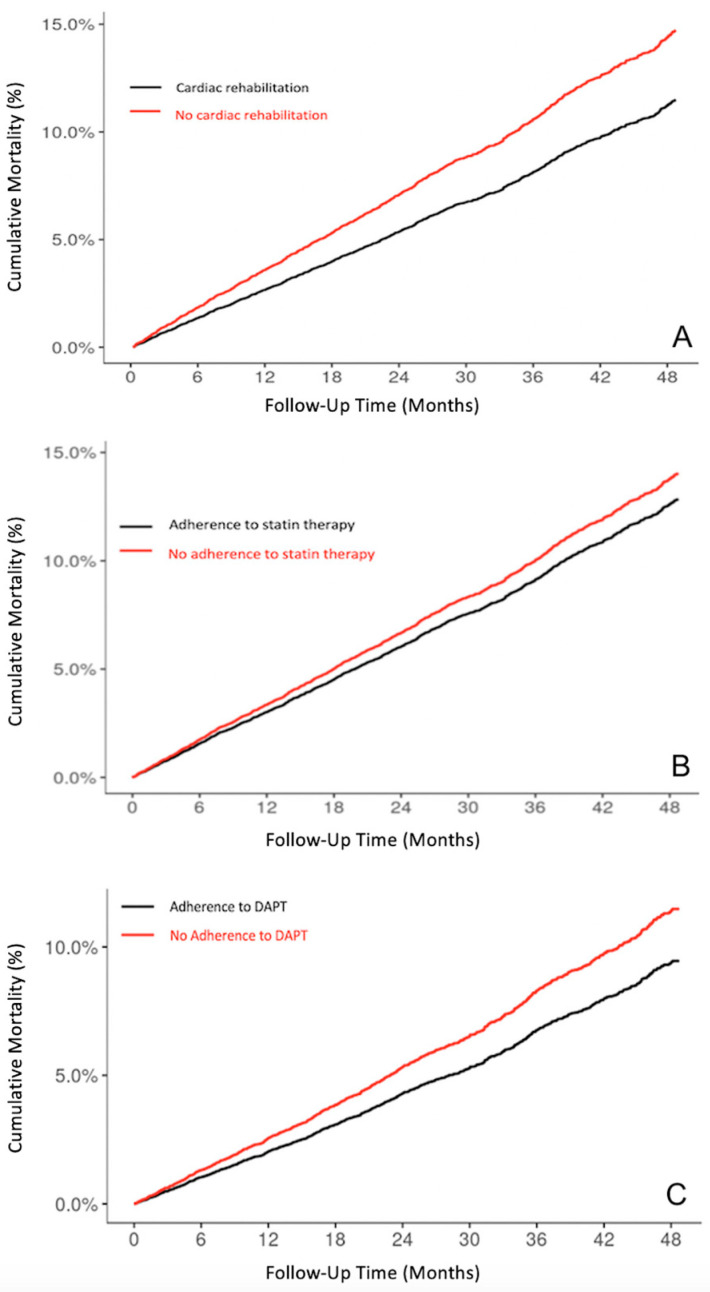
Cumulative mortality comparing individuals with and without adherence to (**A**) cardiac rehabilitation (*p* < 0.001); (**B**) high-intensity statin therapy (*p* < 0.001); (**C**) DAPT (*p* < 0.001). Dual antiplatelet therapy (DAPT).

For the analysis on CR programs, a total of 16,518 patients (median age: 64 years (4–74); male: 68.4%) fulfilled the predetermined inclusion criteria. Notably, an alarming 86.6% (*n* = 14,305) of all individuals diagnosed with acute MI did not engage with any recommended CR programs as outlined in the current guidelines. Throughout the patient follow-up until 01/2018, a total of 1774 individuals (10.7%) died. Adherence to the prescribed CR demonstrated a robust and independent inverse relationship with mortality, exhibiting an adjusted hazard ratio of 0.73 (95% CI: 0.54–0.98; *p* = 0.036); see Figure 1A.

Regarding statin utilization, a total of 23,240 patients (median age: 65 years (55–75); male: 67.7%) met the inclusion criteria. Excluded from the final analysis were individuals who died during the index event (*n* = 366; 1.6%), those who experienced reoccurring myocardial infraction (*n* = 569; 2.4%), or those who were lost during follow-up (*n* = 158; 0.6%). Notably, 66.4% (*n* = 15,422) of all acute MI patients did not adhere to the recommended high-intensity statin therapy as outlined in the current guidelines. The highest incidence of medication discontinuation or termination occurred within the initial month following the index event, accounting for more than 50% of all cases. Throughout the patient follow-up until 01/2018, a total of 3522 individuals (15.2%) died. Non-compliance with high-intensity statins demonstrated a robust and independent association with mortality, with an adjusted hazard ratio of 1.16 (95% CI: 1.06–1.25; *p* < 0.001); see Figure 1B.

In the analysis of DAPT adherence, a cohort of 22,331 eligible patients (median age: 65 years (55–76); male: 68%) were included. Adherence to the guideline-recommended DAPT was found in only 29.3% of patients. Among these, 12.1% showed initial adherence to DAPT within the first months but were then either switched to anticoagulants (79.7%), had recurrent myocardial infarction (14.7%), or died (5.6%). The highest discontinuation rates were found during the first months following the index acute MI event. Overall, 2.3% of all patients died during the observation period. Notably, adherence to DAPT was associated with a 21% mortality risk reduction over the observation period (adj. HR 0.79; 95% CI: 0.72–0.88; *p* < 0.001); see Figure 1C.

## 4. Discussion

The multifaceted therapy approach of CR has been shown to not only reduce mortality but also decrease hospital readmissions and healthcare costs, while simultaneously improving exercise capacity and enhancing quality of life [2]. However, despite clear guideline recommendations, the referral and acceptance of CR frequently fail to reach optimal levels [8]. The findings of the present nationwide analysis also highlighted this discrepancy in adhering to the recommended CR programs subsequent to acute MI. Strikingly, a mere 13.4% of patients demonstrated attendance to CR. Notably, engaging in CR after acute MI was found to be significantly associated with a 27% risk reduction in mortality during the observation period. This finding aligns with prior research conducted in the field [4]. The risk reduction seems to be mediated through addressing lifestyle and risk factor modification during CR [2]. Furthermore, adherence to the recommended medical therapy in patients following acute MI is related to a risk reduction in mortality of at least one-third alone [10]. Therefore, patient education about the nature of their diseases and the importance of the established medical therapy is another crucial aspect of CR. Previous research has already highlighted this trend of increased adherence to pharmacotherapy after CR participation [11].

In conjunction with the observed low CR attendance, the discontinuation of high-intensity statins in about two-thirds of patients was found in the present analysis. Interestingly, 50% of all discontinuations happened in the initial month following the index event. This was linked to a 16% increase in mortality. Generally, the discontinuation of statins is a significant factor in the failure to achieve LDL-cholesterol goals. The DA VINCI observational study, which assessed lipid-lowering therapy in both secondary and primary prevention across Europe, found that only 13% of patients at very high cardiovascular risk achieved their guideline-directed LDL-C targets [12]. Similarly, data from Norway underscore poor adherence to lipid-lowering therapy, with 71% of patients experiencing gaps or discontinuation within a year, and after two years, adherence dropped to just 19% [13]. The primary reasons for discontinuing statins or not adhering to them include statin-associated adverse effects, misinformation about statin therapy, and inadequate patient education leading to fears of side effects [14]. While non-adherence is often blamed on patients, an equally significant factor is physician inertia, which affects both general practitioners and cardiologists [14]. It is noteworthy that statin intolerance is relatively rare, with a global prevalence estimated at around 9.1% based on a comprehensive meta-analysis [15]. The condition is often overestimated, and careful assessment using recognized international definitions is essential to avoid the unnecessary discontinuation of this effective therapy.

DAPT is especially important in the first 12 months following acute MI to prevent further ischemic events and stent thrombosis in the case of percutaneous intervention. However, within the present analysis, 70.3% of patients did not adhere to the prescribed DAPT. This finding was associated with increased mortality of 21%. Non-adherence to DAPT is a well-known key predictor of worse outcomes following coronary stenting [16]. A 2014 systematic review reported discontinuation rates as low as 42.8% [17]. However, these rates have significantly improved over time, likely due to increased awareness and the implementation of clinical guidelines. In a more recent analysis, one-year DAPT discontinuation rates were estimated at 22.8%. Adherence to DAPT was associated with an adjusted hazard ratio of 0.52 compared to premature discontinuation [18]. Besides excessive bleeding risk, the need for surgery that cannot be postponed, complex polypharmacy, the fear of side effects, or the lack of perceived benefit might play an important role in adherence. Furthermore, bleeding events such as bruising and nosebleeds, often labeled as “nonmajor” in clinical trials, can have a profound impact on patients [19]. These events may not only necessitate unexpected medical consultations for bleeding assessments, potentially involving interventions, but can also lead to the discontinuation of antithrombotic therapies. It is plausible to hypothesize that these experiences could contribute to reduced adherence to DAPT, as patients might become increasingly hesitant to continue the treatment due to concerns about recurring bleeding episodes. Therefore, patient education becomes crucial, and this is effectively facilitated by CR programs, which can provide patients with the necessary knowledge and support to manage side effects and adhere to prescribed therapies.

The concerning lack of adherence to essential secondary preventive pharmacotherapy observed in this analysis highlights the urgent need to enhance awareness of CR. Limited participation in CR programs, which are crucial for educating patients on the importance of adherence to prescribed therapies, is a significant issue. Additionally, social, geographical, and economic disparities along with challenges such as limited flexibility in patients’ work lives likely contribute to variations in adherence. Patients in remote areas or those facing financial challenges may experience greater difficulties in accessing healthcare services, potentially leading to higher discontinuation rates. Unfortunately, we were unable to differentiate between inpatient and outpatient CR in this study; however, it is likely that adherence was much higher in outpatient CR programs. By increasing awareness and providing comprehensive education on the benefits of CR, healthcare providers and patients can collaborate more effectively to enhance adherence rates, address socioeconomic barriers, and optimize patient outcomes through more effective risk factor modification strategies.

## 5. Conclusions

In conclusion, the attendance rate for CR observed in this nation-wide study was notably low. Furthermore, patient adherence to high-intensity statins and DAPT was found to be suboptimal, with a significant proportion discontinuing the treatment within the first month of the index MI event. These findings of non-adherence were additionally observed to be significantly associated with an increased risk of mortality. There is a pressing need for increased awareness regarding the benefits of CR and the importance of sustained adherence to secondary preventive medication regimens. Efforts should be directed towards promoting broader awareness among healthcare providers and patients to ensure appropriate referral and participation in CR programs. Moreover, it is essential to establish a structured referral system that addresses potential barriers on multiple levels, as this is crucial to bolster patient attendance rates in CR programs and enhance medical therapy adherence. In addition, implementing regular and personalized follow-up sessions enables healthcare providers to assess medication adherence, address any concerns or obstacles, and offer ongoing support and education to patients. This comprehensive approach, combining patient empowerment facilitated through CR and structured follow-up, might yield substantial improvements in outcomes and overall cardiovascular health.

## Data Availability

Data are available via the corresponding author on reasonable request.
